# Comparison of Water Absorption and Drying in Distal Radius Fracture Casts and Orthoses

**DOI:** 10.5435/JAAOSGlobal-D-21-00115

**Published:** 2021-09-13

**Authors:** David Edward White, Michael John van Wyk

**Affiliations:** From the BioDesign Lab, School of Engineering, Computer and Mathematical Sciences, Auckland University of Technology, Auckland, New Zealand.

## Abstract

**Introduction:**

Traditional plaster and fiberglass casts are not waterproof. This experimental study compares the water-resistant and drying properties of two commercially available orthoses with traditional cast liners.

**Methods:**

Two orthotic brace systems were selected for comparative waterproof testing with plaster and fiberglass traditional cast liners. This entailed water submersion for 10 seconds, followed by light drip drying for another 10 seconds. Moisture levels were then measured at four different locations immediately after drip drying and then every 15 minutes up to 45 minutes.

**Results:**

The Zero-Cast Wx orthosis retained the least moisture after initial immersion and was fully dry within 45 minutes. The Exos upper extremity brace also demonstrated a low initial mean moisture content but lost little moisture during drying. In comparison, both the cotton-lined plaster cast and Delta Dr. cast liner systems demonstrated the greatest amount of water absorbed and moisture retention.

**Discussion:**

Both orthotic brace systems demonstrated markedly less water absorption compared with the cotton-lined plaster cast and Delta Dr. cast liner systems. The Zero-Cast Wx was the only orthosis to fully dry in 45 minutes.

**Conclusion:**

Both orthotic brace systems provide superior water-resistant properties to traditional cotton-lined plaster cast or fiberglass Delta Dr. cast liner systems.

## Distal Radius Fracture Casts

Distal radius fracture (DRF) is the most common fracture seen in the developed world.^1^ The traditional treatment protocol usually involves the wrist and forearm being stabilized for 4 to 6 weeks using plaster or fiberglass casting. Patients with DRF treated with either of these casts are instructed to keep the cast dry to avoid structural damage and to prevent skin and odor problems associated with excess moisture.^2,3^ Patients have reported that this places a burden on daily tasks such as showering, bathing, or hand washing.^4^ A recent study on a pediatric population has shown that nearly 50% of unplanned cast changes can be attributed to casts getting wet, imposing a clinical and financial burden.^5^

Alternative materials such as the Gore-Tex–based cast liner have been developed to address problems associated with wetting of traditional cotton lining materials. Gore-Tex cast liners have been proven to be successful in maintaining function while improving hygiene and reducing unscheduled cast changes.^6^ Although a waterproof liner may be a viable solution, they are known to be expensive and difficult to apply.^7^ One commercially available waterproof liner (Delta Dry) requires 90 minutes to dry off after water exposure.

## Specialized DRF Orthoses

In recent years, other product solutions have entered the market. These are specialized DRF orthoses, usually made from lightweight, breathable, and water-resistant materials. Although some manufacturers of these devices claim their product to be waterproof, to the best of the authors' knowledge, no published studies have assessed water-resistant properties and user experience. This study investigates the water-resistant and drying properties of various DRF treatment devices with the aim of providing patients and clinicians with moisture measurements associated with immersion and drying for various DRF treatments.

The orthotic brace systems in this study have different construction materials. The Exos consists predominantly of thermoplastic and neoprene, whereas the Zero-Cast is injection-molded plastic and memory foam. Although a very small volume of stainless steel metal is present in the two orthoses, in the form of buckles, securing screws and adjusters, both orthotic brace systems are predominantly made of plastic.

## Treatment Cost

It has been noted in a previous study that the unit cost of traditional DRF treatment method (orthosis and plaster cast) represents a small proportion of overall treatment cost.^8^ Although the unit cost of plaster and fiberglass cast materials is cheaper than most other treatments, all direct and indirect costs should be taken into consideration to enable a true cost comparison. It was also noted in the earlier cost study that rehabilitation is one of the largest cost components for DRF injury treatment.^8^

An accurate comparison of material cost and reimbursement is made difficult because the costs of plaster cast materials/treatment are reimbursed using different parameters to the orthotic devices/treatments. The cost of treatment for the plaster and fiberglass materials described in this study is reimbursed using the Healthcare Common Procedure Coding System (HCPCS) Q-code system for casting materials range between $9 and $21 per cast.^9^ In addition, a clinician is reimbursed for applying plaster and fiberglass casts, and actual freight and logistics charges, through Current Procedural Terminology (CPT) codes. In 2014, the actual costs reported charged for fitting short-arm casts across 1,249 unique providers averaged $221.79 (minimum cost $63 and maximum cost $875).^10^

Reimbursement codes assigned to orthotics used for treatment vary depending on the adjustability, materials, injury indications, type of application, and construction design of the device. There may also be variation in codes dependent on the applying professional's qualifications. The orthotic devices described in this study are routinely reimbursed using HCPCS L-codes,^9^ noting that that L-code reimbursement includes costs for the healthcare professional's fitting time, freight/logistics, and follow-up. Although the unit price of these orthotic devices varies and orthotic companies do not widely distribute details of their pricing, it is estimated that the orthotic devices in this study are sold to providers at around $90 to $220 each.

## Measuring Moisture Content

Moisture meters are used primarily to determine moisture content in building materials such as wood, concrete, and plaster; however, they can also be used on nonbuilding materials such as fabrics, insulators, and leathers.^11^ Almost all moisture meters are calibrated to measure wood moisture content and therefore provide a reading called percentage wood moisture equivalent (%WME). The %WME is a theoretical value that would be reached by a piece of wood in contact and in moisture equilibrium with the material of interest. With the range of %WME levels being correlated with moisture content, shown in Table 1, this scale is also a good indicator of the moisture level of any other material.^12-14^

**Table 1:** Classification of Moisture Levels for Fuller Moisture Meter 730-2005

Classification	%WME
Low moisture content	5-11.9
Medium moisture content	12-15.9
High moisture content	16-50

This experimental study compares the water absorption and drying properties of two commercially available orthoses with traditional cast liners to determine whether they offer any functional benefits in improved water resistance compared with traditional casts.

## Methods

### Selection and Comparison of DRF Orthoses

**Figure 1:**
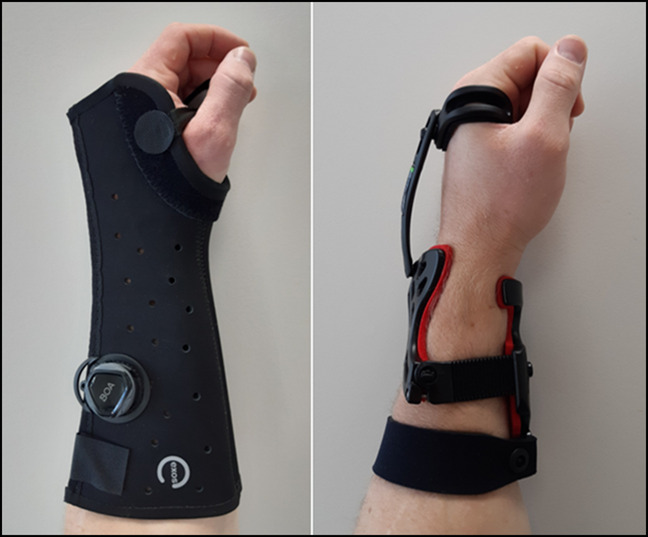
Photographs showing Exos upper extremity brace (left) and Zero-Cast Wx (right).

Two commercially available DRF orthoses, shown in Figure 1, were selected for water absorption and drying testing. The Zero-Cast Wx by Surgisplint is a foam-lined dynamic orthosis that is claimed to be waterproof and breathable.^15^ The Exos upper extremity brace by DJO is claimed to be a waterproof solution for common wrist fractures.^16^ In addition to the two selected orthoses, a cotton-lined plaster cast and the Delta Dr. cast liner were also tested for comparison.

Plaster casts and Exos thermoplastic splints are both moldable; hence, the ability to maintain fracture alignment depends on the applicator's molding skills. It is noteworthy that a definitive study providing evidence of what defines a “good” plaster cast cannot be found in the current literature. Zero-Cast is a prefabricated “off-the-shelf” device available in four sizes that can be adjusted. It claims to provide predictable 3-point fixation at the fracture site, thereby stabilizing the fracture; however, currently, no published clinical fracture alignment data on this device exist.

### Moisture Level Measurement

**Figure 2:**
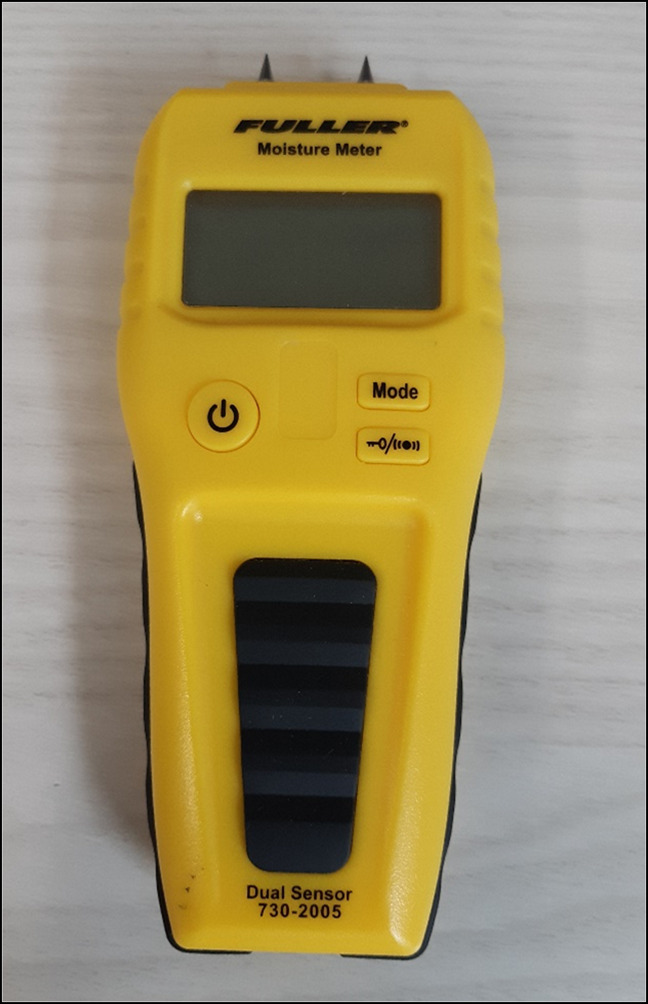
Photograph showing Fuller moisture meter, model 730 to 2005 used in experiment.

**Figure 3:**
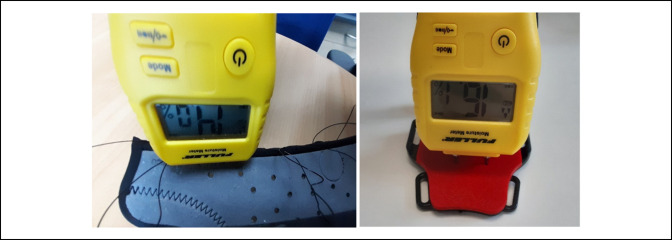
Demonstration of moisture readings being taken on the Exos upper extremity brace (left) and Zero-Cast Wx (right).

A portable two-pin Fuller Moisture Meter 730-2005, shown in Figure 2, was used to measure the moisture content of the skin contact surfaces of the selected DRF treatment devices. The pin-type meter was chosen for cost-effectiveness and ease of use and was calibrated using the calibration circuit board built into the covering cap. Measurement entailed inserting the two steel probes into the material of interest enabling an electric current to pass between the probes. Moisture content, displayed as a %WME reading and detected as a function of measured electrical resistance, was measured on each orthosis by placing the pins of the meter on the surface in contact with the wrist and forearm, shown in Figure 3.

### Experimental Procedure and Data Collection

**Figure 4:**
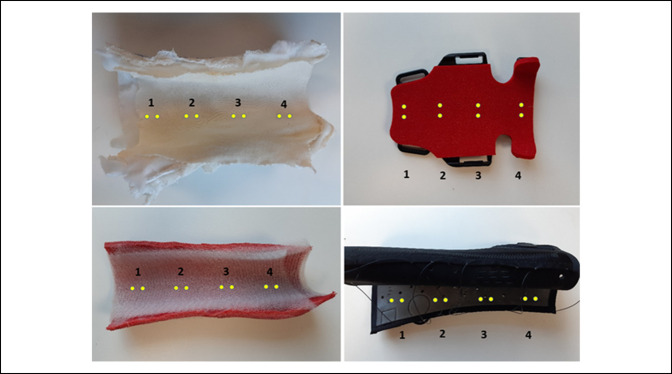
Photographs showing locations of averaged moisture measurements for each orthosis—from top left to bottom right: cotton-lined plaster, Zero-Cast Wx, Delta-Dry, and Exos.

The DRF orthosis, cast liner, and cotton-lined plaster casts were submerged in a water-filled plastic crate for 10 seconds before being drip-dried for a further 10 seconds. This step was intended to simulate a worst case scenario where the splint is accidentally wetted during bathing, showering, or hand washing. Moisture readings were recorded at four sites at least 1.5 cm apart along the skin contact surface for the cotton-lined plaster cast, Delta-Dr. cast liner, Exos upper extremity brace, and Zero-Cast Wx orthosis, as shown in Figure 4. From these four sites, an average %WME value was then calculated to obtain a mean moisture value for each sample.

Moisture levels were recorded immediately after 10 seconds of drip-drying (T = 0) and every 15 minutes thereafter (T = 15) for a total of 45 minutes (T = 45). The experiment was repeated 10 times for each system under test in an environmentally controlled room with a mean air temperature and humidity of 24.45°C (SD = 0.63°C) and 54.78% (SD = 3.2%), respectively.

## Results

**Figure 5:**
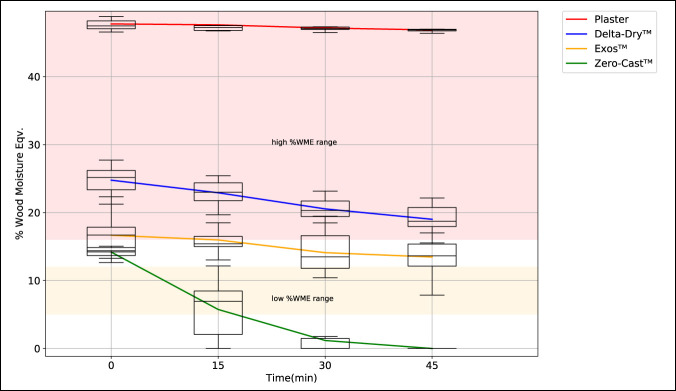
Composite time series plot showing moisture levels of selected DRF orthoses after water immersion.

**Figure 6:**
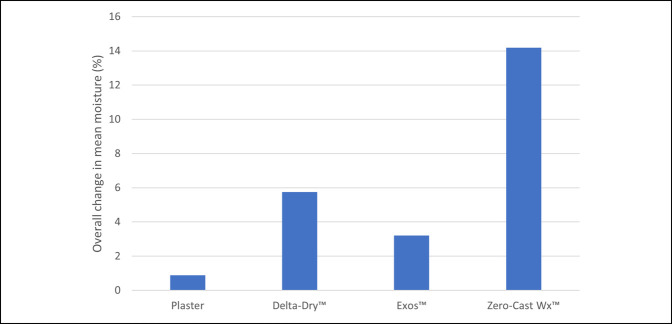
Bar chart showing final changes in the mean % wood moisture equivalent for each orthosis.

The composite time series plot shown in Figure 5 presents the %WME results for all four samples tested. For each measurement time instance (T = 0, T = 15, T = 30, and T = 45), box and whisker plots the distribution of all %WME measurements recorded across 10 repeated experiments. Each colored line plots the mean %WME value for each orthosis or DRF cast system at each time instance. The bar chart in Figure 6 shows the overall change in mean %WME for each orthosis over the 45-minute period and provides an indication of their relative drying rates.

Initial post water immersion moisture content levels varied between all samples with the cotton-lined plaster cast and Delta Dr. cast liner displaying the highest absorption, 47.76% and 24.77 %WME, respectively, compared with Exos upper extremity brace, and Zero-Cast Wx orthosis, at 16.67% and 14.19 %WME, respectively. Forty-five minutes after immersion, the mean %WME value of the cotton-lined plaster cast was relatively unchanged at 46.87%, whereas both Delta Dr. cast liner and Exos upper extremity brace had reduced to 19% and 13.46 %WME, respectively. In comparison, the moisture content of the Zero-Cast Wx orthosis consistently reduced to 0 %WME after 45 minutes in all tests.

The cotton-lined plaster had the highest initial moisture content (at T = 0) with a mean %WME of 47.76% and demonstrated the slowest drying rate and lowest mean reduction in moisture content (Δ = 0.89% WME) over the 45-minute drying period. The Delta-Dr. cast liner had the second highest initial mean moisture content with a value of 24.77 %WME (at T = 0) and a slightly higher reduction in mean moisture (Δ = 5.75%) over the same drying period.

## Discussion

Wrist fractures affect both young and old. A treatment that involves prolonged splintage may cause a notable burden to patients and adversely affect their daily-living activities. In addition, water avoidance over the typical treatment period (6 week) affects both personal hygiene and leisure activity participation.

Both brace-type orthoses performed better in lower initial water absorption; however, the drying performances between them were very different. The Exos upper extremity brace had an initial mean moisture content of 16.67 %WME (at T = 0) but lost little moisture over the total drying time (Δ = 3.2 %WME). The Zero-Cast system was found to absorb the least amount of moisture, with a mean value of 14.19 %WME at time T = 0, and showed the fastest drying rate, being the only sample to completely dry in the 45-minute period. It was also observed that both cotton-lined plaster cast and Delta-Dr. system remained in the high moisture content range throughout the experiment, whereas Exos and Zero-Cast fell into the medium and low moisture ranges, respectively. The mean change in %WME over the 45-minute drying period is illustrated in Figure 6.

A waterproof cast is a desirable feature in DRF treatment. The range of treatments reviewed in this study showed notable differences in water-resistant properties. Water-resistant cast liners such as Delta Dr. have shown to score better in patient satisfaction.^6^ However, our investigation has shown that the two newer devices (Exos and Zero-Cast) demonstrate superior water-resistant properties over Delta Dry. Lower water retention and faster drying will provide patients with increased satisfaction when engaged in water-related activities. Faster drying will also decrease the risk of skin complications associated with wetting. Our study has shown that the Zero-Cast Wx system absorbed the least amount of water and exhibited the fastest drying rate compared with the Exos upper extremity brace and the Delta-Dr. cast liner. Both Zero-Cast Wx and Exos orthoses may provide clinical cost savings and reduce the resources required to address moisture-related splinting issues compared with the Delta-Dr. cast liner and cotton-lined plaster cast systems.

Further investigation should be conducted to verify water resistance while the device is being worn on a patient throughout the entire duration of treatment. Seeking a qualitative evaluation of change in orthosis performance because of wetting, for example, the patient's perception of feeling wet, or the onset of discomfort from skin and odor problems associated with excess moisture, should also be investigated in future work. The devices should also be tested under various ambient conditions to determine the effects of ambient air temperature and relative humidity on drying rates.

## Conclusions

Both orthotic brace systems provide superior water-resistant properties to traditional cotton-lined plaster cast or fiberglass Delta Dr. cast liner systems, and the Zero-Cast Wx was the only orthosis to fully dry in 45 minutes.
